# A Maternal Gene Regulator CPEB2 Is Involved in Mating-Induced Egg Maturation in the *Cnaphalocrocis medinalis*

**DOI:** 10.3390/insects16070666

**Published:** 2025-06-26

**Authors:** Yi Duan, Yueran Xiao, Guo Cai, Kepeng Wang, Chenfan Zhao, Pengcheng Liu

**Affiliations:** 1 Department of Entomology, Nanjing Agricultural University, Nanjing 211800, China; 2022102082@stu.njau.edu.cn (Y.D.); 2023102079@stu.njau.edu.cn (Y.X.); 2024202073@stu.njau.edu.cn (G.C.); 2024102080@stu.njau.edu.cn (K.W.); 12121322@stu.njau.edu.cn (C.Z.); 2 State Key Laboratory of Agricultural and Forestry Biosecurity, College of Plant Protection, Nanjing Agricultural University, Nanjing 211800, China

**Keywords:** juvenile hormone, maternal mRNA, oviposition, reproduction, RNA-binding protein

## Abstract

Cytoplasmic polyadenylation element-binding proteins (CPEBs) are critical regulators of maternal mRNA translation during oogenesis, yet their roles in insect reproduction remain underexplored. Here, we characterized CmCPEB2, a CPEB homolog in the rice leaf roller *Cnaphalocrocis medinalis*, a destructive lepidopteran pest insect, and elucidated its role in mating-induced oviposition. The CmCPEB2 protein harbored conserved RNA recognition motifs and a ZZ-type zinc finger domain and was phylogenetically clustered with lepidopteran orthologs. Spatiotemporal expression profiling revealed *CmCPEB2* was predominantly expressed in ovaries post-mating, peaking at 12 h with a 6.75-fold increase in transcript levels. Liposome-mediated RNA interference targeting *CmCPEB2* resulted in a 52% reduction in transcript abundance, leading to significant defects in ovarian maturation, diminished vitellogenin deposition, and a 36.7% decline in fecundity. The transcriptomic analysis of RNAi-treated ovaries identified 512 differentially expressed genes, with downregulated genes enriched in chorion formation and epithelial cell development. Tissue culture-based hormonal assays demonstrated the juvenile hormone-dependent regulation of *CmCPEB2*, as JH treatment induced its transcription, while knockdown of the JH-responsive transcription factor *CmKr*-h1 in the moths suppressed *CmCPEB2* expression post-mating. These findings established CmCPEB2 as a juvenile hormone-dependent regulator of mating-induced oviposition that orchestrates vitellogenesis through yolk protein synthesis and ovarian deposition and choriogenesis via transcriptional control of chorion-related genes. This study provides novel evidence of CPEB2-mediated reproductive regulation in Lepidoptera, highlighting its dual role in nutrient allocation and structural eggshell formation during insect oogenesis and oviposition.

## Simple Summary

Cytoplasmic polyadenylation element binding protein 2 (CPEB2) is a key protein in regulating oocyte maturation. However, its role in insect reproduction is not fully understood. In this study, a *CPEB2* gene was identified in the rice leaf roller *Cnaphalocrocis medinalis*, an important agricultural pest insect. The analysis at both transcriptional and protein levels showed that CmCPEB2 was highly expressed in the ovaries after male–female mating and was upregulated by the juvenile hormone pathway. The potent knockdown of *CmCPEB2* by liposome-facilitated RNA interference led to abnormal ovarian development and consequently reduced vitellogenin deposition and oviposition. Moreover, the expression levels of the genes associated with eggshells’ formation were significantly downregulated after the knockdown of *CmCPEB2* as revealed by comparative transcriptomics analysis and moth-based assay. Overall, our study suggested that CmCPEB2 plays a crucial role in mating-induced egg maturation in *C. medinalis*.

## 1. Introduction

Oogenesis is a key process in adult female reproduction that includes three stages, previtellogenesis, vitellogenesis, and choriogenesis, according to the status of yolk deposition [1,2]. During vitellogenesis, oocytes absorb vitellogenins and other nutrients from the serum and follicular cells, a process regulated by juvenile hormone (JH) or 20-Hydroxyecdysone (20E) [3,4]. In the course of choriogenesis, follicular epithelium cells initially secrete vitelline membrane proteins, which coalesce into a continuous layer encircling the oocyte. Subsequently, they synthesize chorion proteins, leading to the formation of the eggshell [5,6]. In the reproductive development of *D. melanogaster*, oocytes are arrested at the metaphase stage of the first meiotic division during the growth phase [7]. At this stage, the transcriptional activity of the oocyte is essentially dormant, while the transcription and translation in the nurse cells are highly active, synthesizing maternal mRNAs and proteins [2,8]. At the end of the growth phase, nurse cells undergo a “dumping” process, rapidly transferring all maternal substances to the oocyte [8]. The resumption of the meiosis, maturation, fertilization, and early embryonic development of the oocyte depends on the translational regulation of maternal mRNAs. Therefore, the selective translation and degradation of maternal mRNAs at specific developmental and maturation stages are the key to the success of oogenesis.

In *mouse*, *zebrafish*, *Xenopus laevis*, and *Drosophila*, maternal mRNAs encode key drivers of oogenesis and early embryonic development. They are synthesized during oocyte growth and are immediately deadenylated and stored [9,10,11]. This deadenylation process inhibits the translational activity of mRNAs, placing them in a dormant state within the oocyte. Subsequently, at appropriate developmental stages, these mRNAs can regain their poly(A) tails through cytoplasmic polyadenylation, thereby being reactivated for translation [12]. This mechanism is pivotal in ensuring the timely and orderly expression of maternal mRNAs during oogenesis and early embryonic development [13].

The precise control of maternal mRNA’s translation involves a few key regulators, including cytoplasmic polyadenylation element-binding proteins (CPEBs). CPEBs are a class of highly conserved RNA-binding proteins that mediate the translational control of maternal mRNAs through sequence-specific interactions with cytoplasmic polyadenylation elements (CPEs) in 3′ untranslated regions (3′ UTRs) [12,14]. CPEB proteins include two subfamilies (CPEB1 and CPEB2) across different species. Vertebrates possess four members (CPEB1-4), while *D. melanogaster* retains two (Orb1 and Orb2) [15]. During cytoplasmic polyadenylation, CPEB regulates maternal mRNA via CPE motifs and Maskin interactions [16,17]. The RNP complex integrates antagonistic PARN deadenylase and Gld2 poly(A) polymerase activities: PARN maintains short poly(A) tails and suppresses translation by blocking eIF4E-eIF4G binding [15,18]. Hormone-induced CPEB phosphorylation triggers PARN dissociation, enabling CPSF to recruit poly(A) polymerase for tail elongation. Poly(A)-bound PABP then bridges eIF4G, displaces Maskin, and initiates translation [19,20,21].

Orb1 facilitates the localization of maternal transcripts in ovarian and early embryonic tissues during oogenesis and embryogenesis [22]. The *Orb1* gene is essential for early oogenesis to form 16-cell cysts and later drives oocytes’ differentiation. *Orb1* gene mutation causes female sterility [23,24]. *Drosophila Orb2* establishes oocytes’ polarity and fate and is crucial for long-term memory and stable synaptic specificity [25,26,27].

*Cnaphalocrocis medinalis* (*Lepidoptera*: *Crambidae*), also known as the rice leaf roller, is a prevalent rice pest that poses a significant risk to food security in various parts of Asia. It exhibits strong reproductive capacity: adults mate two days post-emergence and lay eggs a day after mating, enabling a rapid population growth that threatens rice production [28]. Despite the above, the role of CPEBs in insect oogenesis remains elusive. In this study, we demonstrated the regulatory role of CPEB2 in mating-induced egg maturation during reproduction in this agricultural pest insect, thus shedding light on the molecular mechanisms underlying insect reproduction.

## 2. Materials and Methods

### 2.1. Insect Rearing and Sample Preparation

*C. medinalis* were reared under laboratory conditions using wheat seedlings as food in an artificial climate chamber. The conditions were 26 ± 2 °C and a 70 ± 5% relative humidity under a 14:10 dark–light cycle. To maintain the stable growth and development of *C. medinalis*, males and females were placed separately into plastic cups containing cotton balls moistened with a 10% honey solution post adult eclosion. After 48 h, males and females were paired in a plastic cup covered with plastic wrap, and eggs were laid in the film. All the collected samples were kept at −80 °C. All experiments were independently conducted in triplicate, with three biological replicates included for each sample group.

### 2.2. Identification of CmCPEB2 and Bioinformatic Analysis

The CmCPEB2 sequences derived from the transcriptome data in our lab were aligned with InsectBase 2.0 (http://v2.insect-genome.com/ (accessed on 1 June 2025)). A complete open reading frame (ORF) of CmCPEB2 was then amplified via PCR. The purified PCR product was subsequently ligated into pCE2 TA/Blunt-Zero plasmids using the 5 min™ TA/Blunt-Zero Cloning Kit (Vazyme Biotech, Nanjing, China), and the construct was sequenced by Sangon Biotech (Shanghai, China). The amino acid sequence of CmCPEB2 was analyzed using DNAMAN software (version 6.0). Its molecular weight (MW) was predicted by ExPASy (https://web.expasy.org/compute_pi/, accessed on 3 April 2025). The conserved domains within the protein were identified using InterPro online software (https://www.ebi.ac.uk/interpro/search/sequence/, accessed on 9 April 2025) and Illustrate for Biological Sequences (IBS) software (https://www.ibs.renlab.org/#/home, accessed on 23 April 2025). The protein’s tertiary structure was predicted through homology modeling using SWISS-MODEL (https://swissmodel.expasy.org/, accessed on 21 April 2025), with A0A7E5X622_TRINI from the AlphaFold Database serving as the primary template (sequence identity: 99.06%). Validation of the model via Ramachandran plot analysis revealed that 84.91% of residues were located in favored regions, indicating a robust stereochemical quality. To examine the phylogenetic relationships of *CPEB2* genes across different species, the complete amino acid sequences of CmCPEB2 orthologs from 20 other insect species were retrieved from the GenBank database (https://www.ncbi.nlm.nih.gov/genbank/, accessed on 22 April 2025) for the construction of a phylogenetic tree. Multiple sequence alignment was performed using DNAMAN software (https://www.dnaman.net/, accessed on 23 April 2025), and the phylogenetic tree was constructed using the neighbor-joining method in MEGAX (https://www.megasoftware.net/, accessed on 23 April 2025), applying the Poisson correction model, pairwise deletion for gaps, and 1000 bootstrap replicates to assess nodal support.

### 2.3. RNA Isolation and cDNA Synthesis

Total RNA was extracted using TransZol reagent (TransGen Biotech, Beijing, China) according to the manufacturer’s instructions. The purity and concentration of RNA were assessed with a NanoDrop One Micro UV–Vis spectrophotometer (Thermo Fisher Scientific, New York, NY, USA). First-strand cDNA was synthesized from 3 μg of RNA using the HiScript III First-Strand cDNA Synthesis Kit (Vazyme Biotech Co., Ltd., Nanjing, China) equipped with a gDNA wiper, following the manufacturer’s instructions. The synthesized cDNA from each sample was then stored at −80 °C for subsequent general PCR, qRT-PCR, and dsRNA synthesis.

### 2.4. Spatial-Temporal Expression Analysis of CmCPEB2

Adult females of *C. medinalis* were dissected 12 h before and after mating, and samples were collected from eight different tissues: head, thorax, midgut, fat body, Malpighian tubes, ovaries, bursa copulatrix, and carcass. Different stages during ovarian development (0–48 h post-eclosion; 0–24 h post-mating; and age-matched virgins) were collected and stored separately at −80 °C. Each sample consisted of three biological replicates. Total RNA was extracted from pooled replicates and reverse-transcribed into cDNA using the kit described in Section 2.2. All cDNA samples were diluted fourfold and subsequently utilized as templates for qRT-PCR analysis. The reactions were performed using an Applied Biosystems QuantStudio 5 Real-Time 96-well PCR System (Thermo Fisher Scientific, New York, NY, USA) in a 10 μL reaction mixture containing 5 μL 2X SYBR Green *Pro Taq* HS Premix with High Rox Plus (Accurate Biology, Hunan, China), 1 μL of cDNA template, 0.2 μL of each gene-specific primer (Table S1), and 3.6 μL of nuclease-free water. The reaction procedures were set as follows: 96 °C for 5 min, followed by 40 cycles of 96 °C for 15 s and 58 °C for 25 s. Relative expression levels were calculated using the 2^−ΔΔCT^ method with *ribosomal protein L13* (*RPL13*) as the reference gene. Three biological and technical replicates were performed for each sample.

### 2.5. Liposome-Facilitated RNA Interference of CmCPEB2

To investigate the function of *CmCPEB2* in the ovarian development of *C. medinalis*, the RNA interference (RNAi)-based knockdown of *CmCPEB2* gene expression was performed. The target sequence was first amplified by PCR using cDNA as the template, with primers lacking T7 promoter sequences. The purified PCR product was then ligated into the pIEx-4 plasmid. After plasmid amplification in bacterial culture, the extracted plasmid was used as a high-concentration template for dsRNA synthesis through in vitro transcription with T7 RNA polymerase. *GFP* dsRNA, which served as a negative control, was synthesized in vitro from a template amplified from the pIEx-4-GFP plasmid. Both dsRNAs against *CmCPEB2* and *GFP* were synthesized using the MEGAscript™ T7 Transcription Kit (Thermo Fisher Scientific, New York, NY, USA) according to the manufacturer’s instructions. Specific primers for ds*CmCPEB2* and ds*GFP*, each containing a T7 promoter sequence, were designed (listed in Table S1). The synthesized ds*RNAs* were diluted to 4000 ng/µL with RNase-free water and then mixed with prepared liposomes (RFect Plasmid DNA Transfection Reagent, BIOG, Changzhou, China) to achieve a final concentration of 2000 ng/µL.

For the RNAi experiment, thirty newly emerged adult female *C. medinalis* moths were cold-anesthetized and randomly divided into three groups (10 individuals per group) for subsequent experimental treatments. dsRNA injection was performed using a 25-gauge (25G) precision microsyringe (Hamilton 700 series), with 0.5 μL of liposome-encapsulated ds*CmCPEB2* injected into the intersegmental membrane between the second and third abdominal segments. This procedure was repeated 24 h after the initial injection. An equal amount of ds*GFP* was injected as a control. Mating was conducted 24–28 h after the second dsRNA injection, and ovaries were dissected and collected 12 h post-mating (36–40 h after the second injection). Ten insects were randomly collected and processed individually to validate preliminary interference efficacy. Effective samples (with >50% interference) were then pooled to form three biological replicates (n = 3). The pooled RNA samples were used for reverse transcription for the subsequent global interference assessment. The efficiency of RNAi was determined by qRT-PCR as described above.

### 2.6. Effect of CmCPEB2 on Ovarian Development and Female Fecundity

To investigate the effect of *CmCPEB2* knockdown on female fecundity, females injected with ds*CmCPEB2* (n = 30) were paired with age-matched males in a clean plastic cup (male-to-female ratio 1:3) to allow normal mating and egg-laying. Once the adults began laying eggs, the plastic cups were replaced with new ones every 24 h, and the number of eggs laid per day on the old cups (single-female fecundity) was recorded. The daily egg counts were summed to calculate the total number of eggs laid by each female during the first two days of oviposition. This approach ensured that the females were laying eggs while interference with *CmCPEB2* expression remained effective. To observe the ovarian morphology, ovaries from females 12 h post-mating under ds*CmCPEB2* and ds*GFP* treatments were dissected under an NSZ-608T stereomicroscope (Novel, Nanjing, China). The ovaries were placed in sterile PBS, and images were captured using Imageview software (https://www.pooher.cn/download/62.html, accessed on 18 April 2025). Ovarian length measurements were taken from the acquired ovarian phenotype images using the measurement tools in Imageview software. Each ovarian specimen was measured eight times. Additionally, the expression levels of *CmVg* in the fat bodies of females mated for 12 h under ds*CmCPEB2* and ds*GFP* treatments were assessed. Three independent biological replicates were performed for this analysis.

### 2.7. Western Blot Analysis

To examine the ovarian expression pattern of the CmCPEB2 protein, ovarian tissue was dissected from mated adult females and age-matched virgins at various developmental stages (n = 3). Proteins were extracted using an optimized RIPA buffer system supplemented with protease and phosphatase inhibitors (1 mM PMSF, 1× protease inhibitor cocktail, 1× phosphatase inhibitor) on ice for 30 min, followed by centrifugation at 12,000× *g* for 15 min at 4 °C to collect the supernatant. Protein concentrations were determined using the BCA Protein Quantification Kit (Vazyme Biotech, Nanjing, China) according to the manufacturer’s instructions. Proteins were then separated by 10% SDS-PAGE and transferred onto a PVDF membrane. The membrane was probed with a CmCPEB2 antibody (1:5000 dilution, GenScript, Nanjing, China). After washing, an HRP-conjugated goat anti-rabbit IgG polyclonal antibody (ABclonal, Nanjing, China) diluted 1:5000 was applied. Chemiluminescent detection was performed using a Chemiluminescence Detection Kit (Shanghai Tanon Technology Co., Ltd., Shanghai, China), and imaging was carried out with a fully automated multifunctional image analysis system (Tanon). A recombinant anti-GAPDH antibody (1:5000 dilution, Servicebio, Wuhan, China) was used as an internal control. To determine RNAi efficiency at the protein level, females were injected with ds*CmCPEB2* immediately after eclosion, mated for 12 h, and ovarian samples (n = 3) were collected to compare the protein levels of CmCPEB2 and CmVg between ds*CmCPEB2-* and ds*GFP*-injected insects. The anti-CmVg polyclonal antibody, custom-produced by GenScript Biotech Co., Ltd. (Nanjing, China), was used at a working dilution of 1:5000 for immunoblotting. A quantitative densitometry analysis of protein bands was performed using ImageJ software (https://imagej.net/ij/download.html, accessed on 22 April 2025) with background subtraction and normalization to the internal control.

### 2.8. Hormonal Culture and Upstream Regulator Analysis

20E and JH-III (Shanghai Yuanye Bio-Technology Co., Ltd., Shanghai, China) were dissolved in ethanol and stored at −20 °C until use. Fat bodies and ovaries attached to the abdominal body wall were dissected from newly emerged female *C. medinalis* by removing the guts. Three biological replicates, each consisting of 9 females, were cultured for each treatment. The ovary-containing abdomen pelts were cultured in 12-well cell culture plates in vitro, as described previously [29,30]. The composition of the hormone-added media is detailed in Table S2, with the final concentration of the hormones set to 5 μM. Ethanol (ETH) was used as a control. The plates were incubated at 28 °C in a biochemical incubator (Ningbo Southeast Instrument Co., Ltd., Ningbo, China) for 3, 6, and 12 h. After incubation, the samples were collected and stored at −80 °C for subsequent analysis.

RNA was extracted from the collected samples and reverse-transcribed for qRT-PCR analysis. The expression levels of the downstream response factors *CmKr-h1* and *CmHR3* in the JH and 20E pathways were first quantified to assess the impact of hormone treatment. Subsequently, the expression level of *CmCPEB2* was also analyzed. The primers used for these analyses are listed in Table S1. To further investigate the upstream regulators of *CmCPEB2*, we interfered with *Kr-h1*, a transcription factor in the juvenile hormone signaling pathway, and observed changes in *CmCPEB2* transcript levels. ds*CmKr-h1* high-purity template synthesis was performed following the previously described method. ds*CmKr-h1* and ds*GFP* were synthesized using the MEGAscript™ T7 Transcription Kit (Thermo Fisher Scientific, New York, NY, USA) according to the manufacturer’s instructions. The specific ds*Cmkr-h1* primers containing a T7 promoter sequence were designed and are listed in Table S1. Ovary tissues were collected 12 h after the secondary injection of ds*CmKr-h1* to evaluate the efficiency of gene knockdown. All the experiments were performed with three biological replicates.

### 2.9. cDNA Library Preparation and Illumina Sequencing

cDNA library preparation and Illumina sequencing were conducted at Benagen Technology Co., Ltd. (Wuhan, China) using next-generation sequencing (NGS) technology. Approximately 12 ovaries from *C. medinalis* females at 12 h post-mating were collected from each treatment group (ds*CmCPEB2*-treated) and the control group (ds*GFP*-treated) following dsRNA ingestion. Each treatment consisted of three biological replicates. RNA concentration and purity were measured using the Qubit RNA Assay Kit with a Qubit 3.0 Fluorometer (Life Technologies, Carlsbad, CA, USA). A total of 1 μg of RNA was used to construct the cDNA library with the NEBNext^®^ UltraTM RNA Library Prep Kit for Illumina^®^ (New England Biolabs, Ipswich, MA, USA), following the manufacturer’s instructions. The prepared cDNA library was sequenced by the Illumina Novaseq 6000 platform (Illumina, San Diego, CA, USA), generating paired-end reads of 250–300 bp. Raw reads underwent quality control using fastp (v0.21.0) with default settings: Phred score ≥20, minimum length 36 bp, and automated adapter removal. Filtered reads were further assessed for quality through secondary QC using FastQC (v0.11.9), to evaluate base quality distributions and sequence complexity. Additionally, the Q20, Q30, and GC content of the clean data were calculated.

### 2.10. Transcriptome Analysis After Knockdown of CmCPEB2

In the absence of a reference genome for the transcriptomic data, de novo assembly was performed using Trinity (version 2.11.0; parameter: –min_kmer_cov 2) to reconstruct transcripts from the clean reads. This approach generated a splice junction database based on the gene model annotation file. The expression levels of these genes were calculated using reads per kilobase of exon per million reads mapped (RPKM). Differential expression analyses of genes between the ds*GFP* and ds*CmCPEB2* groups were performed using the DESeq2 R package(v1.26.0) [31]. Significance filtering was performed using adjusted *p*-values (padj < 0.05 and |log2FoldChange| > 1). If the number of significantly differentially expressed unigenes (DEUs) was insufficient, the threshold was adjusted to *p*-value < 0.05 and |log2FoldChange| > 1. Functional enrichment analyses of DEGs, including Gene Ontology (GO) pathway analyses and Kyoto Encyclopedia of Genes and Genomes (KEGG) pathway analyses, were conducted using clusterProfiler (version 3.14.3). All *p*-values were corrected for the testing of multiple hypotheses, and the adjusted *p*-values (qvalue) ranged within [0, 1].

### 2.11. Data Analysis

All statistical analyses and data plots were performed using GraphPad Prism 8.0 (https://www.graphpad.com/scientific-software/prism/, accessed on 18 April 2025). Prior to statistical analysis, the normality and homogeneity of variance were assessed using the Kolmogorov–Smirnov test. Two-way ANOVA was applied to assess differences in means among multiple groups to determine if significant differences existed. One-way ANOVA followed by Tukey’s post hoc test was used to evaluate differences in female fecundity. Student’s *t*-test was employed to compare the means between two independent samples and determine statistical significance. *p*-values below 0.05 were considered statistically significant (* *p* < 0.05, ** *p* < 0.01, *** *p* < 0.001, **** *p* < 0.0001).

## 3. Results

### 3.1. Sequence Characterization and Phylogenetic Analysis of CmCPEB2

**Figure 1:**
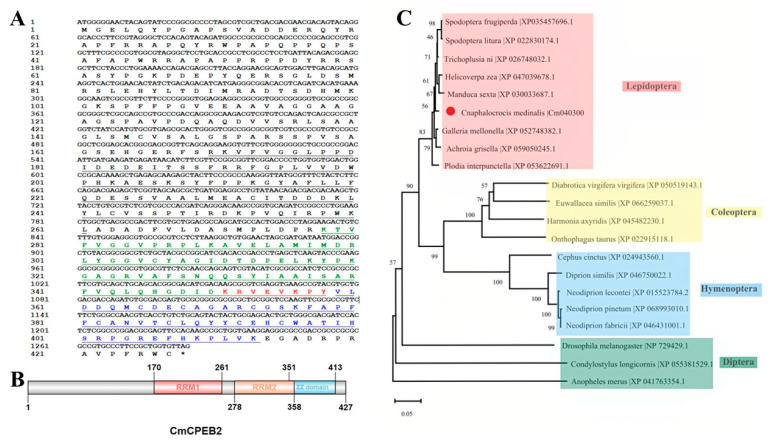
Bioinformatics analysis of CmCPEB2. (**A**) The nucleotide and amino acid sequences of the *CmCPEB2* gene are presented. Numbers on the left indicate nucleotide and amino acid positions. The black solid line represents the RNA recognition motif 1 (RRM1), while the red font and underlined text highlight the overlapping region between the RNA recognition motif 2 (RRM2, shown in green) and the ZZ domain (shown in blue). (**B**) Structural domain analysis of the CmCPEB2 protein. (**C**) Phylogenetic analysis of CPEB2 proteins across different insect species. CmCPEB2 is marked with a red dot. The GenBank accession numbers for each species are listed on the tree. The phylogenetic tree was constructed using the neighbor-joining method in MEGAX, with bootstrap support calculated from 1000 resampled data sets.

Based on the genome data in the online database (InsectBase 2.0, http://v2.insect-genome.com/, accessed on 18 April 2025) and the transcriptome data from our laboratory, this study characterized the gene *CPEB2* in *Cnaphalocrocis medinalis* (Lepidoptera: Crambidae) (CmCPEB2, GenBank accession number: PV550082). The identified CmCPEB2 contains a 1284-bp open reading frame (ORF) encoding a 427-amino acid polypeptide with a predicted molecular mass of 47.12 kDa and an isoelectric point (pI) of 6.72 (Figure 1A).

Domain architecture analysis revealed evolutionary conservation at the C-terminus, featuring two RNA recognition motifs (RRM1: aa 170–261; RRM2: aa 278–358) and a ZZ-type zinc finger domain (aa 351–413) (Figure 1B). Notably, the ZZ domain exhibits partial overlap with the RRM2 domain, suggesting potential functional interplay between these structural elements.

**Figure 2:**
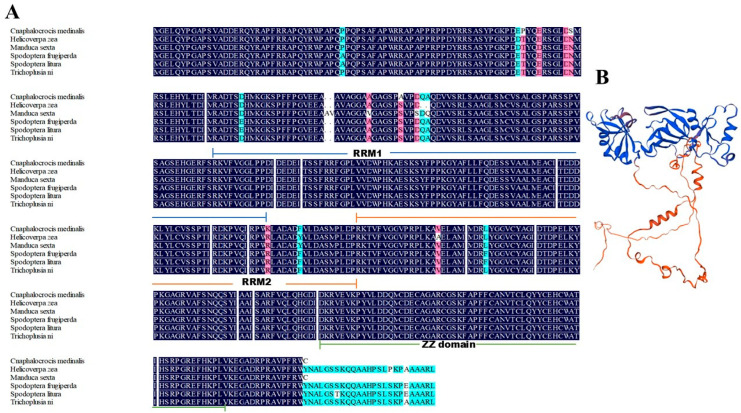
Multiple sequence alignment and protein structure analysis of CmCPEB2. (**A**) Multiple sequence alignment of CPEB2 proteins from various lepidopteran insects, including *Cnaphalocrocis medinalis*, *Helicoverpa zea*, *Manduca sexta*, *Spodoptera frugiperda*, *S. litura,* and *Trichoplusia ni*. The color-coded sequence identity annotations are defined as follows: blue indicates 100% identity, red denotes identity ≥75%, and green represents identity ≥50%. (**B**) Protein structure analysis of CmCPEB2 performed using SWISS-MODEL software.

Phylogenetic reconstruction using CPEB2 homologs from diverse insect species demonstrated that CmCPEB clusters within the lepidopteran clade, showing the closest evolutional relationships to *Trichoplusia ni* (Noctuidae), followed by *Spodoptera litura*, *S. frugiperda*, *Helicoverpa zea*, and *Manduca sexta* (Figure 1C). Comparative sequence analysis revealed a high conservation of CmCPEB2 with these lepidopteran orthologs, sharing a 96.66% pairwise amino acid identity (Figure 2A). In contrast, more distant relationships were observed with dipteran and coleopteran homologs. Tertiary structure prediction using SWISS-MODEL further supported functional conservation, revealing a canonical RRM-ZZ domain topology characteristic of CPEB family proteins (Figure 2B).

### 3.2. Ovary-Specific and Mating-Induced Expression of CmCPEB2

**Figure 3:**
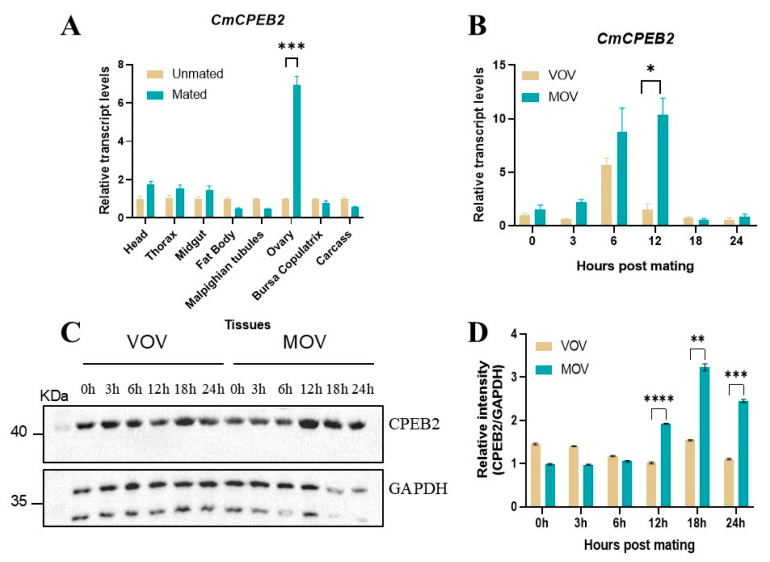
Spatiotemporal expression analysis of *CmCPEB2* in *C. medinalis*. (**A**) Tissue-specific expression analysis of *CmCPEB2* in virgin and mated females. Various tissues were dissected at 12 h post-mating, with age-matched virgin tissues serving as controls. (**B**) Developmental stage expression analysis of *CmCPEB2* in virgin and mated ovaries. VOV refers to virgin ovaries; MOV refers to mated ovaries. The 2^−ΔΔCt^ method was adopted to calculate the relative expression level. (**C**) Representative Western blot images showing CmCPEB2 expression in mated ovaries compared to age-matched virgin ovaries (n = 3). GAPDH antibody was used as a loading control (the upper band). (**D**) Quantitative analysis of CmCPEB2 immunoblot signal intensity. Data are expressed as mean ± standard deviation (SD). Statistical analysis was performed using a two-way ANOVA; asterisks indicate the significance differences (* *p* < 0.05, ** *p* < 0.01, *** *p* < 0.001, **** *p* < 0.0001).

To investigate the role of *CmCPEB2* in insect reproduction, the spatiotemporal expression profile of *CmCPEB2* was investigated across various developmental stages and tissues of *C. medinalis* using qRT-PCR. The results revealed ubiquitous *CmCPEB2* expression in all the examined tissues, including the head, thorax, midgut, fat body, Malpighian tubes, ovary, bursa copulatrix, and carcass. Notably, the mated ovaries exhibited significantly elevated expression levels of *CPEB2* compared to age-matched unmated controls (*p* < 0.001, two-way ANOVA; Figure 3A).

Time-course analysis demonstrated the dynamic regulation of *CmCPEB2* in post-mating ovaries, with transcript abundance peaking at 12 h post-mating, representing a 6.75-fold increase relative to unmated individuals (*p* < 0.5, two-way ANOVA; Figure 3B). To further confirm the above results, Western blotting was employed to assess CmCPEB2 expression at the protein level. The results revealed a corresponding temporal expression pattern, with a maximal protein abundance observed at 12 h post-mating (Figure 3C). Densitometric quantification confirmed significantly higher CmCPEB2 protein levels in mated ovaries compared to unmated controls (*p* < 0.01, two-way ANOVA; Figure 3D). This finding suggests that *CmCPEB2* may play a crucial regulatory role in the reproductive development of the ovaries in *C. medinalis*.

### 3.3. Liposome-Facilitated Knockdown of CmCPEB2 Impeded Female Reproduction

**Figure 4:**
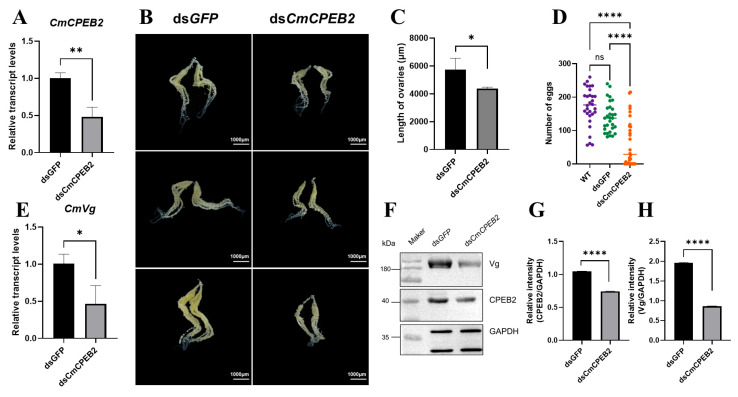
Investigation of *CmCPEB2* function in ovary development via RNAi. (**A**) Quantification of *CmCPEB2* transcriptional levels: *CmCPEB2* expression levels were measured by qRT-PCR following ds*CmCPEB2* or ds*GFP* treatment. (**B**) Effect of *CmCPEB2* knockdown on ovarian development: ovaries were dissected from adult females treated with ds*CmCPEB2* or ds*GFP* at 12 h post-mating. (**C**) Ovarian length (μm) in ds*CmCPEB2*-treated and control groups. Data presented as mean ± SD. (**D**) Fecundity analysis: The effect of *CmCPEB2* knockdown on female fecundity was assessed by counting the number of eggs laid by wild-type and ds*GFP*- and ds*CmCPEB2*-injected females (n = 30). (**E**) *CmVg* expression in the fat body: Relative expression levels of *CmVg* in the fat bodies of females at 12 h post-mating after *CmCPEB2* or *GFP* dsRNA injection were quantified. (**F**) Western blotting analysis of CmVg in ds*GFP-* and ds*CmCPEB2*-injected mated ovaries. The antibody against GAPDH was used as a loading control (the upper band). (**G**) Quantification of immunoblot signal intensity (CmCPEB2) after *CmCPEB2* knockdown. (**H**) Quantification of immunoblot signal intensity of CmVg after *CmCPEB2* knockdown. The 2^−∆∆Ct^ method was adopted to calculate the relative expression level. Data are expressed as mean ± standard deviation (SD). The asterisks indicate significant differences (* *p* < 0.05, ** *p* < 0.01, **** *p* < 0.0001).

To investigate the functional role of *CmCPEB2* in ovarian development, systemic RNA interference (RNAi) was performed using liposome-encapsulated double-stranded RNA (dsRNA). qRT-PCR analysis confirmed significant transcript depletion (a 52% reduction, *p* < 0.01, Student’s *t*-test) in ds*CmCPEB2*-injected females compared to ds*GFP*-injected controls at 12 h post-mating (36–40 h after the second injection), validating effective gene knockdown (Figure 4A).

Phenotypic characterization revealed pronounced reproductive defects in ds*CmCPEB2*-treated females. Ovaries exhibited marked morphological abnormalities, including a reduced size (a 23.44% decrease in longitude axis, n = 3, *p* < 0.01, Student’s *t*-test) and diminished oocyte maturation compared to controls (Figure 4B,C). Subsequent fecundity assays demonstrated that *CmCPEB2* knockdown significantly impaired egg-laying capacity, with a 36.7% reduction in total oviposition during the first 48 h post-mating (Figure 4D). This indicated that *CmCPEB2* may be significantly involved in promoting oviposition after female mating.

Based on the above, we were intrigued to examine whether knockdown of *CmCPEB2* affected the level of vitellogenin (Vg), one of the yolk precursor proteins and the most abundant protein in the ovary, which is synthesized in the fat body and taken up by the developing oocytes. The results indicated that the transcript level of *CmVg* in the fat body was significantly reduced after ds*CmCPEB2* treatment (Figure 4E), while Western blot detected a corresponding downregulation of CmVg protein in the ovaries (Figure 4F–H). Collectively, these results demonstrated that *CmCPEB2* was involved in ovarian development and vitellogenesis and, ultimately, fecundity in *C. medinalis*.

### 3.4. CmCPEB2 Was Regulated by the Juvenile Hormone Pathway

**Figure 5:**
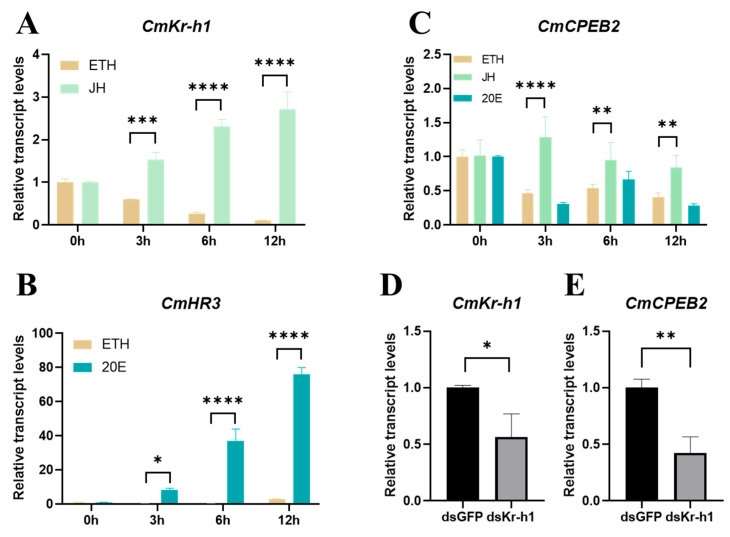
*CmCPEB2* was regulated by the juvenile hormone pathway. Transcriptional dynamics of *CmKr-h1* (**A**) and *CmHR3* (**B**) following hormonal treatment. Ethyl alcohol (ETH) was used as a control. (**C**) Transcription dynamics of *CmCPEB2* in vitro hormonal-cultured ovaries. (**D**) The knockdown efficiency of *CmKr-h1* was validated in ovarian tissue. (**E**) *CmCPEB2* transcriptional response to *CmKr-h1* knockdown. The 2^−∆∆Ct^ method was adopted to calculate the relative expression level. Data are presented as mean ± standard deviation (SD). The asterisks indicate the significant differences (* *p* < 0.05, ** *p* < 0.01, *** *p* < 0.001, **** *p* < 0.0001).

To explore the upstream signaling pathway that governs the elevation in *CmCPEB2* level post-mating, the ovaries were freshly dissected from newly emerged moths and then cultured in vitro in a medium containing juvenile hormone III (JH III) and 20-hydroxyecdysone (20E) at a final concentration of 5 μM. The expression of krüppel homolog 1 (*Kr-h1*), a JH-inducible gene, and hormone receptor 3 (*HR3*), a 20E-inducible gene, was initially examined following hormone treatment. An evident increase in the expression of *CmKr-h1* was observed at 3, 6, and 12 h after JH-III treatment, compared to those at 0 h (Figure 5A). Likewise, the transcriptional upregulation of *CmHR3* was found after the ovaries were cultured in the presence of 20E for 3, 6, and 12 h (Figure 5B). The above samples were then used to investigate the expression of *CmCPEB2*, and the results indicated a significant increase in the transcriptional level of *CmCPEB2* after JH-III treatment at 3, 6, and 12 h. However, no upregulation of *CmCPEB2* expression was found in the 20E-treated samples, irrespective of the treatment’s duration (Figure 5C).

To further confirm the hormonal regulation of *CmCPEB2* expression, we next knocked down *CmKr-h1*, a key transcription factor in the JH pathway, using the RNAi as described in previous sections. The RNAi efficiency in each individual moth at 24 h post the second injection of dsRNA was examined first, and then a genuine pool of samples with at least a 40% knockdown efficiency was obtained (Figure 5D, *p* < 0.05, Student’s *t*-test). The transcription of *CmCPEB2* was investigated in the above samples with qRT-PCR. The results suggested that *CmCPEB2* expression was significantly decreased in the ovary samples after the potent knockdown of *CmKr-h1* (Figure 5E). Taken together, we concluded here that *CmCPEB2* was upregulated by the juvenile hormone signaling pathway.

### 3.5. CmCPEB2 Was Likely Involved in Choriogenesis

**Figure 6:**
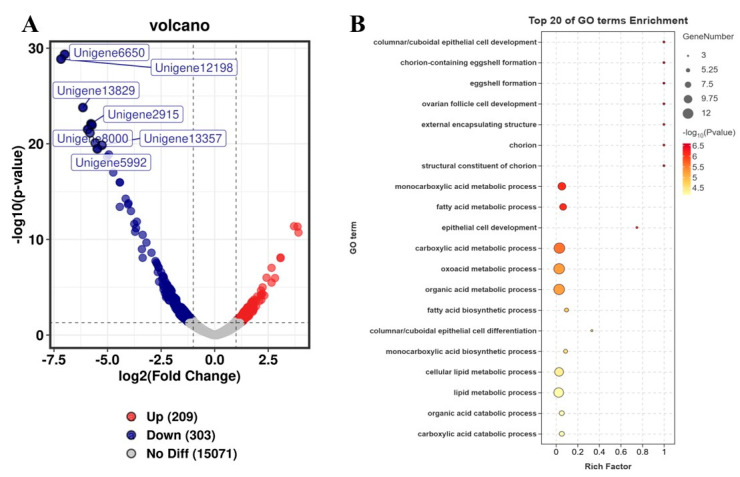
Identification of DEGs between the ds*CmCPEB2* and ds*GFP* groups. (**A**) A volcano diagram for each gene. The red and blue points indicate upregulated genes and downregulated genes, respectively. The gray dots indicate no significant differences. (**B**) GO enrichment analysis of downregulated DEGs between ds*CmCPEB2* groups and ds*GFP* groups. The size of the bubble indicates the number of DEGs enriched to the corresponding term. The color of the bubble indicates the Q value.

**Figure 7:**
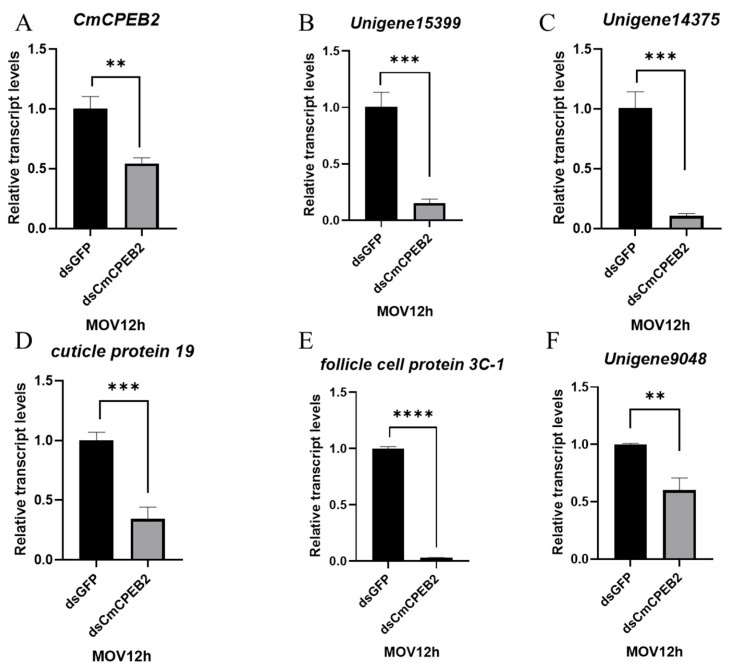
Validation of DEGs involved with chorion-containing eggshell formation. Values are mean ± standard deviation (SD). The 2^−∆∆Ct^ method was adopted to calculate the relative expression level. Significant differences between the ds*CmCPEB2* group and the control group were determined by Student’s *t*-test. The asterisks indicate significant differences (** *p* < 0.01, *** *p* < 0.001, **** *p* < 0.0001).

A depletion of *CmCPEB2* resulted in delayed ovarian development, leading to a subsequent reduction in egg production. This observation prompted us to pursue a deeper study on the physiological role of CmCPEB2 and its underlying mechanisms in the mating-induced egg maturation of *C. medinalis*. According to the above-mentioned method, RNA samples with a valid *CmCPEB2* knockdown were used for the library construction and next-generation sequencing (Figure 6). Transcriptome analysis identified a total of 512 differentially expressed genes (DEGs) (Table S3), of which 209 DEGs were upregulated and 303 DEGs were downregulated in *CmCPEB2* knockdown samples when compared to the ds*GFP*-treated control (Figure 6A). GO enrichment analysis suggested that downregulated DEGs were mainly involved in columnar/cuboidal epithelial cell development and chorion-containing eggshell formation (Figure 6B), which are important for the completion of insect oogenesis. KEGG enrichment revealed that downregulated DEGs were associated with the AMPK signaling pathway and fatty acid metabolism (Figure S1). A total of 12 genes related to choriogenesis were subjected to further analysis, and five were found differentially expressed with biological significance, while the rest were not (Table 1). These five genes were then selected, and their relative transcript levels were examined by qRT-PCR. The results showed that the expression of unigene15399, unigene14375, unigene9048, cuticle protein 19, and follicle cell protein 3C-1 were largely affected upon the effective knockdown of *CmCPEB2* when compared to the ds*GFP*-treated control, which was consistent with the RNA-seq analysis (Figure 7). Therefore, we reasoned here that *CmCPEB2* regulated choriogenesis during oogenesis, thus playing a critical role in mating-induced egg production in *C. medinalis*.

## 4. Discussion

In this study, we characterized the *CPEB2* gene from the lepidopteran pest insect *C. medinalis* and elucidated its role in mating-induced oviposition. The gene was upregulated by the juvenile hormone signaling pathway upon mating, which was a prerequisite to the accumulation of vitellogenin in the rapidly developing oocytes and to eggshell formation before oviposition.

Sequence analysis revealed that CmCPEB2 contains two RRM motifs and a ZZ-type zinc finger domain at the C-terminus. Comparative analysis revealed that *Drosophila* Orb2 isoforms (Orb2A/Orb2B) and CmCPEB2 share conserved RNA-binding domains (RBDs) and C-terminal zinc fingers essential for RNA–protein interactions [32,33]. Notably, *Drosophila* Orb2 contains an N-terminal glutamine-rich (Q) domain that drives the amyloid-like aggregation required for the consolidation of synaptic memory [34]; this functional domain is absent in CmCPEB2. Phylogenetic analysis further demonstrated that lepidopteran CPEB2 proteins form a distinct clade with a high sequence conservation, suggesting the evolutionary conservation of core molecular functions.

Our findings indicated that *CmCPEB2* exhibits a predominant expression in the ovaries of *C. medinalis* peaking at 12 h post-mating. Meanwhile, vertebrate *CPEB2* displays a dual expression in ovarian and spermathecal tissues [35]. In *Drosophila*, *Orb1* is restricted to the germline and the early embryo’s pole cells [23,36,37], whereas *Orb2* is widely expressed in both somatic and germline cells, with the highest levels detected in the embryonic, larval, and adult central nervous system (CNS) as well as in the germ cells of male testes [38,39]. Notably, despite these divergent expression patterns, the conserved enrichment of CPEB2/Orb2 orthologs in reproductive tissues, whether in lepidopteran ovaries (CmCPEB2), dipteran testes (*Drosophila* Orb2), or murine gonadal tissues, suggests an evolutionarily conserved role in gametogenesis.

Liposome-encapsulated dsRNA-mediated gene knockdown in *C. medinalis* adults achieved a significant transcript depletion of *CmCPEB2* (>50%), which correlated with a severely reduced ovary size, diminished egg production, and decreased vitellogenin deposition in the ovaries. These findings were in alignment with studies in other species, highlighting the critical role of CPEB2 orthologs in reproductive development. In insect reproduction, developing oocytes acquire a large amount of vitellogenin (Vg) to meet nutritional needs for egg maturation. Through qRT-PCR, we found that *CmVg* biosynthesis in the fat body was significantly reduced after *CmCPEB2* knockdown, while Western blot analysis revealed a corresponding decrease in its protein level in the ovaries. Rouhana L et al. showed that genes related to yolk gland development were downregulated and yolk deposition became abnormal after interfering with *CPEB2* in *Schmidtea mediterranea* [40]. CPEB activation requires certain hormones in different species. While progesterone-induced CPEB1 activation in *Xenopus* oocytes occurs through Aurora A-mediated phosphorylation and insulin-driven PI3 kinase signaling [21], our findings suggested that *CmCPEB2* responded to juvenile hormone treatment in *C. medinalis*. Therefore, we speculate that JH may activate *CPEB2* in the moths, though direct evidence of JH-driven *CPEB2* activation, including biochemical validation of ligand–receptor binding and a downstream signaling cascade, is still lacking. Given the changing hormone dynamics in different insects, our results here may differ or be limited by species or different developmental stages.

Chorion formation, the final stage of insect oocyte development, involves follicle cells depositing chorion proteins onto the oocyte surface to form the eggshell [41]. Micropyle formation occurs as micropyle cells retract during ovulation, creating openings for sperm entry [42]. Transcriptomic profiling revealed that the liposome-facilitated knockdown of *CmCPEB2* significantly suppressed chorion-related gene expression in ovaries 12 h post-mating. Transcriptome sequencing identified 12 egg formation-related DEGs, 5 of which were significantly downregulated in ds*CmCPEB2*-treated ovaries compared to controls. Of these, follicle cell protein 3C-1 (fcp3c-1) has been reported to be associated with chorion formation in the brown planthopper *Nilaparvata lugens* [43]. Mutations in *Orb* also lead to the production of ventralized eggshells [44]. In *Drosophila*, the *gurken* gene, regulated and localized by *Orb*, influences the secretion pattern and structural assembly of eggshell proteins by regulating the dorsal–ventral polarity of follicle cells. Disruptions in its function cause eggshell defects, mimicking missing dorsal micropyles or overdeveloped ventral areas [45,46,47]. We speculate that inhibiting *CmCPEB2* could severely disrupt eggshell formation and Vg deposition, thereby affecting the reproduction of the rice leaf roller.

## 5. Conclusions

In summary, we identified a *CPEB2* gene in the pest insect *C. medinalis*. *CmCPEB2* exhibited a high expression in the ovaries at 12 h post-mating, which depended on the juvenile hormone signaling pathway. The inhibition of *CmCPEB2* with liposome-encapsulated dsRNA led to an abnormal developmental phenotype, most likely by affecting the expression and deposition of CmVg. Moreover, the comparative transcriptome sequencing analysis and qRT-PCR of *CmCPEB2* RNAi ovaries collectively indicated a suppressed expression of key genes involved in chorion-containing eggshell formation. This study provides novel insights into understanding the role of RNA-binding proteins in reproduction in *C. medinalis*.

## Figures and Tables

**Table 1:** Differentially expressed genes associated with eggshell formation.

Gene ID	Gene Description	Log2FC
Unigene14375	chorion class CB protein M5H4-like	−3.3596
Unigene15399	chorion class CB protein M5H4-like	−2.6874
Unigene9048	chorion class CB protein M5H4-like	−1.14
Unigene5435	follicle cell protein 3C-1	−3.7529
Unigene8510	cuticle protein 19	−1.6402
Unigene16156	putative cuticle protein CPH43	0.8625
Unigene17732	flexible cuticle protein 12-like	0.2520
Unigene9909	larval cuticle protein LCP-17-like	−0.2457
Unigene6040	histone-lysine N-methyltransferase 2B-like, partial	−0.0795
Unigene11771	pupal cuticle protein 20-like	0.0296
Unigene2691	protein naked cuticle homolog	0.1379
Unigene12583	cuticle protein 14	0.0134

Note: The bolded transcripts were validated using qRT-PCR.

## Data Availability

The RNA-seq datasets generated during this study have been deposited in the NCBI Sequence Read Archive (SRA: https://www.ncbi.nlm.nih.gov/sra, accessed on 22 April 2025) under BioProject accession number PRJNA1254169. These data will be publicly available after the indicated release date.

## Supplementary Materials

The following supporting information can be downloaded at: https://www.mdpi.com/article/10.3390/insects16070666/s1, File S1: KEGG enrichment analysis of downregulated DEGs (Figure S1), dsRNA targeting sequences; Table S1: Primers used in this study; Table S2: In vitro medium formulations; Table S3: Identification of significantly differentially expressed genes in different treatments; Table S4: GO enrichment analysis of DEGs; Table S5: KEGG enrichment analysis of DEGs.
