# Supplementary material for: A Maternal Gene Regulator CPEB2 Is Involved in Mating-Induced Egg Maturation in the Cnaphalocrocis medinalis
Source: Insects. 2025 Jun 26;16(7):666. doi: 10.3390/insects16070666 (PMC12295579; doi:10.3390/insects16070666)
Supplement: Supplementary file 1 [file insects-16-00666-s001.zip › Supplementary Materials.pdf]

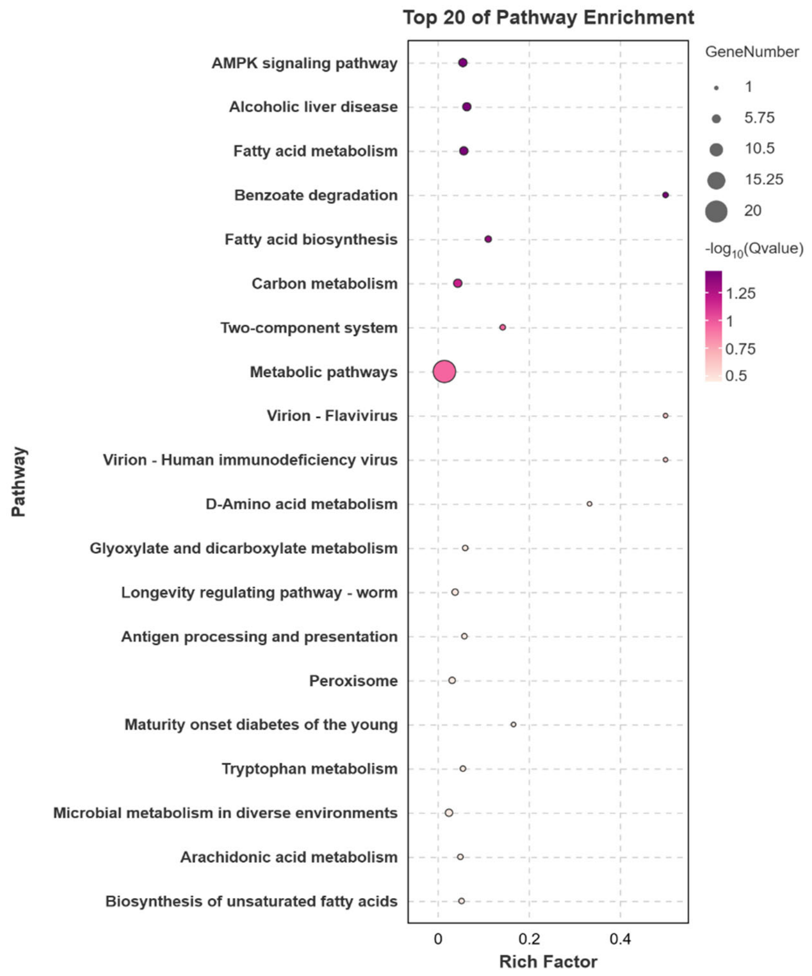

Figure S1. KEGG enrichment analysis of downregulated DEGs between ds*CmCPEB2* groups and ds*GFP* groups. The size of the bubble indicates the number of DEGs enriched to the corresponding term. The color of the bubble indicates the Q value.

dsRNA target sequence and size

ds*CmCPEB2*: 570bp

Target sequence:

AAGCTGTACCTGTGCGTCTCGTCGCCCACGATCAGGGACAAGCCGGTGCAGATCCGGC
CCTGGAAGCTGGCTGACGCCGACTTCGTGCTGGACGCCAGCATGCCACTGGACCCTAG
GAAGACTGTCTTTGTGGGAGGCGTGCCGCGTCCTCTTAAGGCTGTGGAACTAGCGATG
ATAATGGACCGGCTGTACGGCGGCGTCTGCTACGCCGGCATCGACACCGACCCTGAGC
TCAAGTACCCGAAGGGCGCGGGGCGCGTGGCGTTCTCCAACCAGCAGTCGTACATCG
CGGCCATCTCCGCGCGCTTCGTGCAGCTGCAGCACGGCGACATCGACAAGCGCGTCG
AGGTGAAGCCGTACGTGCTGGACGACCAGATGTGCGACGAGTGCGCGGGCGCGCGCT
GCGGCTCCAAGTTCGCGCCGTTCTTCTGCGCCAACGTCACCTGTCTGCAGTACTACTG
CGAGCACTGCTGGGCGACGATCCACTCTCGGCCCGGACGCGAGTTCCACAAGCCGCT
GGTGAAGGAGGGCGCCGACCGCCCGCGCGCCGTGCCCTTCCGCTGGTGTTAG

ds*CmKr-h1*: 692bp

Target sequence:

GAGCGACCCTTTGAATGTGAATATTGTCACAAGATGTTCAGTGTCAAAGAAAACTTGC
AGGTGCATCGCCGTATCCACACCAAAGAAAGACCTTATCGATGCAACGTATGCAATGCT
GCCTTCGAACACTCAGGGAAACTTCATCGTCATGCTCGCATCCACACTGGCGAGAGAC
CTCACGCGTGCCCGCACTGTCACAAACGTTCATTCAATCCGGACAGTTGGTCATCCAC
TTGCGGACGCACACTGGTGAAAAGCCCTATCGTGTCCAGCTCCAGGATGTGGAAAGG
GATTTACCTGTTCGAACAATTAAAGGTGCACTCGCGCACTCACACTGGAGAGAGACCG
TACACTTGCGACATTTGTCTCCGTGATTTCGGCTACAACCACGTTTTGAAGCTGCATCG
CTTCCAACATTACGGCGAACGCTGCTACCGCTGCACTGTATGTGATGGAACATTTAATA
CCAAAAGCAAATGGAGGCTCACATATACAAAGAACATGGTGCCTGAAACTCCTCGCGT
GACTCCAGTGCAATCTTCGGTACCTATTGTAGTTAACGGAAACGTTATGTGTGACCTCG
TGGAGGCGGCACTGCAGCAGCTTCCGCCCACTCCCCCAAGCTCACCTCCATCCCCACC
ATGTGGTGCGCCGCAGGCTGTGCCATCTGAGACATCATCAACTTCGTCGCC

ds*GFP*: 506bp

Target sequence:

ATGGTGAGCAAGGGCGAGGAGCTGTTCACCGGGGTGGTGCCCATCCTGGTCGAGCTG
GACGGCGACGTAAACGGCCACAAGTTCAGCGTGTCCGGCGAGGGCGAGGGCGATGCC
ACCTACGGCAAGCTGACCCTGAAGTTCATCTGCACCACCGGCAAGCTGCCCGTGCCCT

GGCCCACCCTCGTGACCACCCTGACCTACGGCGTGCAGTGCTTCAGCCGCTACCCCGA
CCACATGAAGCAGCACGACTTCTTCAAGTCCGCCATGCCCGAAGGCTACGTCCAGGAG
CGCACCATCTTCTTCAAGGACGACGGCAACTACAAGACCCGCGCCGAGGTGAAGTTC
GAGGGCGACACCCTGGTGAACCGCATCGAGCTGAAGGGCATCGACTTCAAGGAGGAC
GGCAACATCCTGGGGCACAAGCTGGAGTACAACTACAACAGCCACAACGTCTATATCA
TGGCCGACAAGCAGAAGAACGGCATCAAGGTGAACTTCAAGATCCG
