# Supplementary material for: A Maternal Gene Regulator CPEB2 Is Involved in Mating-Induced Egg Maturation in the Cnaphalocrocis medinalis
Source: Insects. 2025 Jun 26;16(7):666. doi: 10.3390/insects16070666 (PMC12295579; doi:10.3390/insects16070666)
Supplement: Supplementary file 1 [file insects-16-00666-s001.zip › Table S1 Premier list.pdf]

Table S1. Primers used in this study

Purpose	Name	Sequences（5'-3'）
RT-qPCR	RPL13-QF	GTGCAACAACCAGCTCCTAA
	RPL13-QR	ACGTGCTCCTCTCAGGTATT
	CmCPEB2-QF	GCCCAAGGGTTATGCGTTTC
	CmCPEB2-QR	CAGGTACAGCTTGTCGTCGT
	CmKr-h1-QF	CAAAGAACATGGTGCTGAAACT
	CmKr-h1-QR	CCTCCACAAGGTCACACATAA
	CmHR3-QF	AGCTCACTCCAACACCAATC
	CmHR3-QR	CAGTCCAGCCACATCTCTTC
	Unigene15399-QF	GTCCTCGACTCGACTCCTCT
	Unigene15399-QR	CCAGGTAGTTCTCGGAAGCG
	Unigene14375-QF	ATCAAGCACCCGCTACAGTC
	Unigene14375-QR	CTACTGCCAAGACTCAGCCC
	Unigene5435-QF	GCTGTTGGATCCTTCTGGCT
	Unigene5435-QR	AGTCCGCATCTTCTTCCGTG
	Unigene8510-QF	CCATGGCGACTACTATGCGT
	Unigene8510- QR	CACGAGTCTCCCACTGTGTC
	Unigene9048-QF	GGACTTGGTCTCGGCTTCAA
	Unigene9048-QR	GTTGGCGTTGCCAAGGTTAC
PCR	CmCPEB2-F	ATGGTCCATGGGGGAACTACA
	CmCPEB2-R	ACACCAGCGGAAGGGCA
RNAi	DsGFP-F	<u>TAATACGACTCACTATAGGG</u>ATGGTGAGCAAGGGCGAGGAG
	DsGFP-R	<u>TAATACGACTCACTATAGGG</u>CGGATCTTGAAGTTCACCTTG
	DsCmCPEB2-F	<u>GCGTAATACGACTCACTATAGG</u>AAGCTGTACCTGTGCGTCTC
	DsCmCPEB2-R	<u>GCGTAATACGACTCACTATAGG</u>CTAACACCAGCGGAAGGGC
	DsCmKr-h1-F	<u>GCGTAATACGACTCACTATAGG</u>GAGCGACCCTTTGAATGT
	DsCmKr-h1-R	<u>GCGTAATACGACTCACTATAGG</u>GGCGACGAAGTTGATGATGT

The black underline indicates T7 promoter.
