# Supplementary material for: A Maternal Gene Regulator CPEB2 Is Involved in Mating-Induced Egg Maturation in the Cnaphalocrocis medinalis
Source: Insects. 2025 Jun 26;16(7):666. doi: 10.3390/insects16070666 (PMC12295579; doi:10.3390/insects16070666)
Supplement: Supplementary file 1 [file insects-16-00666-s001.zip › Table S2.pdf]

Table S2. In vitro medium formulations

Reagent	Volume/Mass
Sf-900™ II SFM (Thermo Fisher Scientific, New York, NY, USA)	10 mL
Penicillin-Streptomycin Solution，100× (Servicebio, Wuhan, China)	100 μL
Glutathione, Reduced (Shanghai RYON Biotechnology Co., Ltd,Shanghai, China)	9 mg
Kanamycin	20 μL
Ampicillin	20 μL
JH-III/20E (Shanghai Yuanye Bio-Technology Co., Ltd., Shanghai, China)	final concentration to 5 μM
